# Mitigating PFAS contamination in the United States: assessing the impact of California’s legislation from 2018 to 2022 on drinking water quality

**DOI:** 10.1057/s41271-025-00594-6

**Published:** 2025-08-23

**Authors:** Sameer D. Bagga, Iris J. N. Parshley, Lindsay Tallon

**Affiliations:** 1 New York University School of Liberal Studies, 726 Broadway, New York, NY 10003 USA; 2 International Research Institute of North Carolina, Durham, NC USA; 3 Massachusetts College of Pharmacy and Health Sciences, Boston, MA USA

**Keywords:** California, PFAS, Drinking water, Policy analysis, Environmental justice

## Abstract

This study evaluates the impact of California’s specific per- and polyfluoroalkyl substances (PFAS) legislation on perfluorooctanoic acid (PFOA) and perfluorooctane sulfonate (PFOS) contamination levels in public drinking water. We conducted a comparative statistical analysis using data collected by the United States Environmental Protection Agency (US EPA) Unregulated Contaminant Monitoring Rules (UCMRs), specifically UCMR 3 and UCMR 5. To assess PFOA and PFOS levels in active public water systems during the pre-legislation period (2017) and the post-legislation period (2023) we applied Levene’s test to assess differences in variances, followed by unpaired and Welch’s *t*-tests to compare mean PFAS concentrations between the two time periods. We detected a significant decline in both PFOA and PFOS levels post-legislation, suggesting that robust state-level regulatory measures can effectively reduce PFAS contamination. Findings highlight the potential for California’s comprehensive approach to serve as a model for national policy to mitigate PFAS exposure and protect public health.

## Key messages

- Rigorous, state-level PFAS legislation may lead to significant reductions in contaminants in public drinking water.
- Robust monitoring, transparent data management, and targeted risk assessments are critical for evaluating the effectiveness of environmental policies.
- Adopting California’s comprehensive regulatory approach at the national level could enhance public health protection and guide sustainable environmental practices.

## Introduction

Amidst the dynamic landscape of the United States (US) public water systems an unsettling reality has come to light. In 2023, PFAS were detected in about 45% of US drinking water samples [1] in water supplies serving over 25 million people. In California, among the 248 active public water systems subjected to testing, 65% have unveiled the presence of per- and polyfluoroalkyl substances (PFAS) within their waters, negatively affecting the lives of more than 16 million residents throughout the state [2]. This high prevalence of PFAS contamination in California has ignited a fervent concern among environmental advocates, health authorities, and policymakers. In response to this escalating crisis, California took proactive measures from 2018 to 2022, enacting legislation to mitigate the adverse repercussions of PFAS on public health and the environment. A troubling revelation in terms of environmental justice is that almost 69% of communities that are identified by the state as disadvantaged are dealing with PFAS contamination in their public water systems [3]. Among these, a quarter contend with the state’s highest levels of PFAS contamination [3].

PFAS, with their innate attributes of waterproofing, grease proofing, and non-stick capabilities, have become ubiquitous in both consumer products and the broader environment. In particular, perfluorooctanoic acid (PFOA) and perfluorooctane sulfonate (PFOS), two of the most used PFAS compounds in various applications [4], are considered long-chain PFAS as they contain eight or more fully fluorinated carbon atoms. This structural characteristic is associated with greater persistence and bioaccumulation in the environment [5]. Due to their desirable functional attributes to repel oil and water, all PFAS, and especially PFOA and PFOS, have permeated diverse sectors of consumer goods, including cookware, waterproof clothing, food packaging, and firefighting foam. The widespread use and persistence of PFAS and the documented links between exposure to these substances and health effects have raised significant concerns. Anchoring this concern are a growing body of epidemiological and toxicological studies that have consistently demonstrated associations between PFAS exposure and carcinogenic effects such as various forms of cancer, including testicular, kidney, and thyroid cancers, immune system dysfunction, liver impairments, developmental and reproductive impairments, and perturbations in hormonal equilibrium [6].

In this paper we aimed to provide a brief review of California’s legislative response and analyze the policies’ subsequent impact on PFAS presence in drinking water and evaluate the efficacy of the multifaceted legislative framework introduced by the state from 2018 to 2020. Central to this examination is a comparative analysis of PFAS levels in drinking water from 2017 to 2023 in California, a region emblematic of the challenges of drinking water contamination.

## Data and methods

### Systematic review of policies

We examined websites and databases from government agencies and organizations actively involved in the analysis, development, and evaluation of PFAS-related policies in California. These activities included monitoring PFAS contamination levels, assessing the efficacy of existing policies, and formulating new regulatory frameworks. These sources include the California Water Boards, Environmental Protection Agency (EPA), California Environmental Protection Agency (CalEPA), Natural Resources Defense Council (NRDC), National Conference of State Legislatures (NCSL), and the PFAS Governance Tracker.

**Fig. 1:**
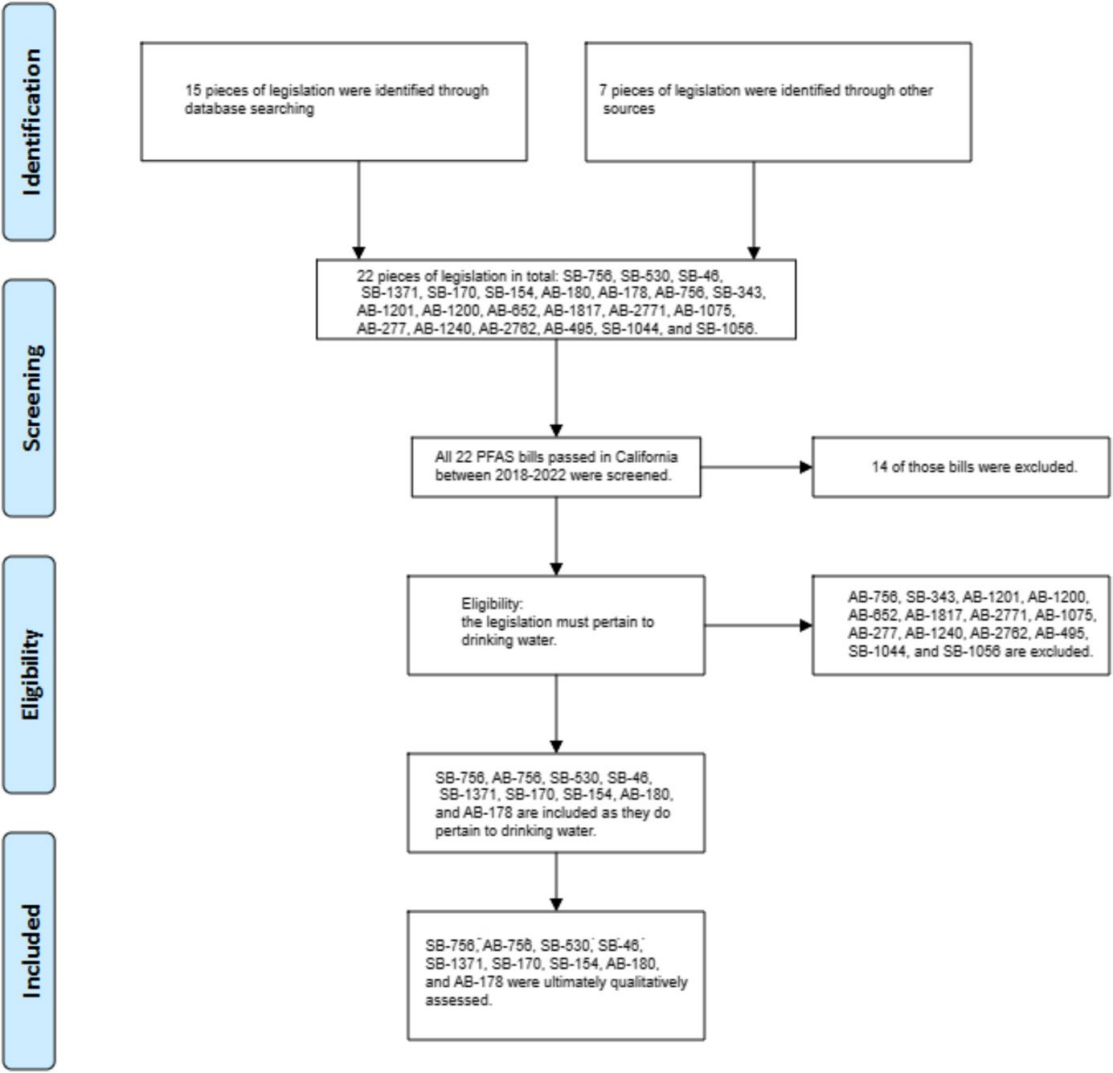
Selection process for a review of policies in California

In order for legislation to be included in our review of California’s regulatory framework concerning PFAS, it had to meet specific inclusion criteria. First and foremost, the legislation had to pertain to drinking water, focusing on regulating, monitoring, or remediating PFAS contamination, specifically PFOA and PFOS, in public and private water systems in California specifically. Additionally, the legislation had to fall within the time period of 2017 to 2023 and have gone into effect between January of 2017 and July of 2023. We initially found 22 potential policies from databases and other sources for our review. After screening based on the inclusion criteria, 14 policies were excluded due to not pertaining to drinking water. The selection process is shown in Fig. 1.

### Measures and PFAS levels

For our analysis, we used data collected from the EPA to investigate water contamination, specifically relying on Unregulated Contaminant Monitoring Rule (UCMR) data, including data from UCMR 3 [7] and UCMR 5 [8]. These data streams are part of the EPA’s Safe Drinking Water Act (SDWA) initiative, which mandates that public water systems (PWSs) monitor unregulated contaminants on a five-year cycle. The guidelines for contamination outlined by the California State Water Resources Control Board were used. “High contamination” for perfluorooctanoic acid (PFOA) was measured as anything above the 0.0051 µg per liter (µg/L) mark. In terms of perfluorooctane sulfonate (PFOS), “high contamination” was anything above the 0.0065 µg/L mark.

To investigate the impact of California’s specific PFAS legislation on PFOA and PFOS contamination levels, we conducted a comparative statistical analysis focusing on their concentrations measured in California’s public water systems in 2017 (pre-legislation period) and 2023 (post-legislation period). The pre-legislation era was deemed to be 2017 and before as the year 2018 marked the onset of significant legislative action, characterized by a large number of bills introduced throughout California addressing PFAS concerns [9]. Thus, the post-legislation era commences from 2018 and after. The study aimed to determine whether the legislation enacted during the post-legislation period had a discernible effect on PFAS contamination. Although other PFAS chemicals may be present, PFOA and PFOS were selected due to their abundance in the environment, harmful health effects, regulatory significance, and the availability of reliable, consistent data for these compounds over the study period.

We compared PFAS contamination levels between the two defined periods using the means and standard deviations; an unpaired t-test was employed to determine if the mean contamination level for each contaminant was significantly different in 2017 versus 2023, and Levene’s test was used to assess whether the differences in variances were significant. Because the EPA tested entirely different sites for PFOA and PFOS in 2017 compared to 2023, with no overlap between the two periods, an unpaired *t*-test was appropriate as long as the Levene’s tests did not produce significant differences in variances between the groups. A Welch’s *t*-test was further conducted to ensure that variance was not an influence on the significance of the result. A statistically significant difference in PFAS contamination levels for each contaminant between the two time periods could suggest that the legislation has had a positive or negative effect on reducing PFAS contamination in drinking water.

Statistical figure generation was conducted using Python v3.12.6 [10], with the matplotlib 3.10.0 [11] and NumPy v2.2.2 packages [12]. Levene’s tests were conducted via the *levene* function from the SciPy v1.15.1 package [13]. Both unpaired and Welch’s t-tests were conducted using the *ttest_ind* function from the SciPy v1.15.1 package [13].

## Results

We found a final total of 8 policies, SB-46, SB-1371, SB-530, SB-756, SB-170, SB-154, AB-180, and AB-178, that matched all selected criteria (Table 1).

**Table 1:** Description of legislative bills included in a systematic review of California policies

Bill name	Date signed into law	Summary
SB-46	October 10, 2019	Aims to regulate the use of PFAS in consumer products and wastewater discharges. The bill requires companies to disclose the presence of PFAS in their products and restrict the use of certain PFAS chemicals in food packaging, carpet cleaners, and other products
SB-1371	September 30, 2020	Requires monitoring of public water systems for PFAS
SB-530	October11, 2020	Provide financial support to water utilities in order to expedite the replacement of drinking water sources that were contaminated with PFAS
SB-756	October 11, 2020	This bill aims to establish maximum contaminant levels (MCLs) for certain PFAS in drinking water, as well as requiring water system testing, reporting, and remediation efforts
SB-170	September 23, 2021	Provides $30,000,000 for technical and financial assistance to drinking water systems to address PFAS
SB-154	June 27, 2022	Allocates $50,000,000 for technical and financial assistance to drinking water systems to address per- and polyfluoroalkyl substances
AB-180	June 30, 2022	Includes $30 million to address per- and polyfluoroalkyl substances in drinking water systems
AB-178	June 30, 2022	Includes $50 million to address per- and polyfluoroalkyl substances in drinking water systems

UCMR 3 and 5 data consisted of contamination levels measured in µg/L. 46 sites were tested for PFOA in 2017 and 25 in 2023, and 60 sites were tested for PFOS in 2017 and 33 in 2023 (Table 2). Our analysis of changes in PFOA and PFOS levels in water revealed a significant decline in average concentrations in both PFOA from 0.028 µg/L to 0.008 µg/L and PFOS from 0.057 µg/L to 0.012 µg/L; Levene’s test indicated that the variances were similar and not significantly different (PFOA: *p* = 0.07; PFOS: *p* = 0.09) (Table 2).

**Table 2:** Descriptive statistics of PFOA and PFOS levels in California in January 2017 and July 2023

	PFOA 2017	PFOA 2023	PFOS 2017	PFOS 2023
Total water sources tested	46	25	60	33
Mean (µg/L)*	0.028	0.008	0.057	0.012
Standard deviation	0.007	0.004	0.023	0.008
Variance**	5 × 10^–5^	2 × 10^–5^	5 × 10^–4^	6 × 10^–5^

^*^Using an unpaired *t*-test, we found the reductions in average concentration to be statistically significant (PFOA: *p* = 1.42 × 10⁻^1^⁸; PFOS: *p* = 7.78 × 10⁻^1^⁸)

^**^Welch’s *t*-test confirmed the results (PFOA: *p* = 2.29 × 10⁻^21^; PFOS: *p* = 1.82 × 10⁻^22^), demonstrating that differences in variance did not affect the significance, as all p-values were well below the 0.05 threshold

## Discussion

The regulation of PFAS in California, as exemplified by policies such as SB-46, SB-1371, SB-530, SB-756, SB-170, SB-154, AB-180, and AB-178 (see Table 1 for descriptions and date of adoption), has been instrumental in addressing the issue of PFAS contamination in drinking water. Primarily, SB-756 set Maximum Contaminant Levels (MCLs) for certain PFAS in drinking water and mandated that water utilities must monitor and take action to reduce PFAS levels in their water sources if they exceed the specified MCLs. This proactive monitoring and remediation can lead to a reduction in PFAS contamination in drinking water. Furthermore, SB-46 aimed to regulate the use of PFAS in consumer products, including food packaging and carpet cleaners. By restricting the use of PFAS in these products and requiring companies to disclose their presence, the potential for PFAS to migrate into the environment and contaminate water sources when these products are disposed of is reduced. Additionally, SB-530, SB-170, SB-154, AB-180, and AB-178 provided financial support to water utilities to expedite the remediation of drinking water sources contaminated with PFAS. Faster remediation means that contaminated sources are taken out of service more quickly and fixed, ultimately reducing water contamination.

Our analysis of the water contamination data obtained from the California State Water Resources Control Board indicates a significant decline in PFAS concentrations between the pre-legislation (2017) and post-legislation (2023) periods—a trend that coincides with the implementation of policies such as SB-756, SB-46, AB-756, and SB-530. However, because our study relies on a comparison of means using unpaired and Welch’s t-tests, we cannot conclusively attribute the observed decline solely to these legislative measures. Other factors—including changes in industrial practices, advancements in water treatment technologies, or natural variations in PFAS levels in water sources—may also have played a role. Although our findings suggest that these policies may have contributed to the observed decline in PFOA and PFOS levels, the lack of PFOA and PFOS contamination data in UCMR 4 [14] (recorded in the years between 2017 and 2023) and compliance data from specific sites research hinders our ability to conduct more rigorous causal inference methods such as interrupted time series, difference-in-differences, and panel data regression analysis. Conducting these analyses could strengthen the relationship between legislative action and water quality improvements.

### Policy recommendations

After evaluating the impact of legislation passed in California from 2018 to 2023 on PFAS water contamination levels, we can see that some progress has been made in addressing this pressing issue. However, there remains room for improvement and the implementation of further measures to ensure continued reduction in PFAS contamination. One promising avenue for future legislation is the establishment of stricter industrial controls for waste streams. Industries that use or produce PFAS-related substances must be held accountable for managing their waste and emissions more responsibly [15]. These controls could include mandatory treatment of industrial wastewater to remove PFAS before discharge and incentives for adopting safer alternatives to PFAS in products. Ultimately, there is a need for a zero-waste hierarchy and a need to push industry toward adopting a circularity model as a path to reducing environmental health harms from PFAS.

Moreover, we recommend that the California state government collaborates with advocacy groups like the Environmental Working Group (EWG) and the Center for Environmental Health (CEH) to strengthen advocacy efforts and ensure that the concerns of residents are heard while passing legislation. In California, EWG has been instrumental in pushing for state-level legislation for greater transparency and stricter standards regarding the presence of harmful chemicals in consumer products, water, and food. They have conducted several studies highlighting issues specific to California. CEH is headquartered in California. Their work in the state involves safeguarding residents from toxic chemicals in various areas, including consumer products and drinking water. Their focus areas include climate and energy, water, urban land use, and conservation of lands and wildlife in the San Francisco Bay Area. By collaborating with these advocacy groups, California can gain a better understanding of the status quo and what its residents truly want, hence improving the quality of the legislation passed.

States like California, with demonstrated commitment to PFAS regulation, can serve as models for other states and the federal government. Under the Safe Drinking Water Act, the EPA has the authority to set enforceable National Primary Drinking Water Regulations (NPDWRs) for drinking water contaminants and require monitoring of public water systems [16]. With this authority, on 10 April 2024, the EPA announced the final NPDWR for six PFAS chemicals: PFOA, PFOS, PFHxS, PFNA, HFPO-DA (commonly known as GenX Chemicals), and mixtures containing two or more of PFHxS, PFNA, HFPO-DA, and PFBS [17]. In our view, the EPA’s regulatory efforts could be further strengthened by adopting some of the more rigorous standards pioneered by California. For example, SB-46 regulated the use of PFAS in consumer products and wastewater discharges. The bill required companies to disclose the presence of PFAS in their products and restrict the use of certain PFAS chemicals in food packaging, carpet cleaners, and other products. By mandating product-level disclosure and imposing targeted restrictions, SB-46 directly curtails the primary pathways through which PFAS enter the environment and reduces consumer exposure to these chemicals. Moreover, if the EPA were to adopt similar measures nationally, it would enable the systematic reduction of PFAS emissions from both industrial and consumer sources, thereby decreasing the overall environmental burden of PFAS and substantially mitigating the associated health risks. In addition to the enforceable standards set under the Safe Drinking Water Act, the Toxic Substances Control Act (TSCA) provides the EPA with critical authority to gather comprehensive data on the manufacture, import, and use of PFAS. Recent TSCA mandates that any entity that has manufactured PFAS in any year since 2011 will need to report their data to EPA through Central Data Exchange (CDX) [18]. This enables the EPA to map out novel and crucial pathways through which these chemicals enter the environment—especially into drinking water systems. With this dataset in hand, the EPA could take further action, similar to its restrictions on polychlorinated biphenyls (PCBs) in 1979 [19], which are also endocrine-disrupting chemicals that can interrupt hormone delivery and circadian rhythm [20, 21], to impose targeted bans or limitations on PFAS uses that are found to contribute significantly to environmental contamination and public health risks.

Building on these state-level successes, cooperation among states and collaboration with the federal government—particularly in Congress and the EPA—can facilitate the development of a comprehensive national framework. As demonstrated by California’s rigorous PFAS regulatory measures, which our analysis shows may have led to significant reductions in PFAS contamination in drinking water, the federal government can adopt similar standards to benefit the nation. National policies modeled on California’s example would provide critical guidance and support to states that have enacted less stringent PFAS legislation. By implementing policies similar to California’s, Congress and the EPA could streamline compliance for industries and utilities, reduce environmental disparities among states, and substantially mitigate the public health risks associated with PFAS exposure throughout the country.

Our study spans a period of several years, allowing for a robust comparison between pre-legislation and post-legislation periods, offering valuable insights into the impact of legislative changes. The study relies on official data collected by the California State Water Resources Control Board, enhancing the reliability and credibility of the findings. The research methodology is transparent and replicable by focusing on specific PFAS chemicals and using established cutoff points for contamination levels. The study’s comparative analysis approach is also a strength. By comparing PFAS contamination levels before and after the introduction of legislation, the study offers a clear indication of the potential impact of regulatory measures on water quality. We had a robust review and policy analysis of relevant legislation. The study goes beyond data analysis to provide valuable policy recommendations for addressing PFAS contamination. It offers a forward-looking perspective on potential avenues for future legislation, which can guide policymakers and stakeholders in addressing this complex issue.

### Limitations

It is essential to acknowledge the potential limitations of the study, including the fact that while testing has covered half of the population in California, as of 2023 it only represents 3% of public water systems in the state [2]. Many small public water systems and private wells are not included in the monitoring program, leaving over 19 million Californians’ water untested for PFAS [2]. This lack of testing means that the actual extent of PFAS pollution in the state is likely far more significant than current data suggests, especially in rural and disadvantaged communities that rely on small water systems or private wells. Moreover, California’s current testing only includes 18 PFAS chemicals, with health advisories established for only three of them. This leaves unknown data for many PFAS chemicals, making it difficult to fully understand the true extent of PFAS pollution and its associated harms [2]. Further research could explore additional factors contributing to PFAS contamination and assess their relative impact on water quality. Seasonal fluctuations may also influence PFAS concentrations in surface and groundwater sources, with increased runoff during wet seasons or lower dilution during dry spells potentially affecting contaminant levels. Without accounting for these temporal variations, the observed differences between 2017 (measured in January) and 2023 (measured in July) may partly reflect seasonal dynamics.

### Implications

The reduction in PFAS concentrations observed in our study may have important public health and economic ramifications. Lower levels of PFOA and PFOS in drinking water may decrease the population’s overall exposure to these harmful substances. Epidemiological studies have linked PFAS exposure to adverse health outcomes, including certain cancers, immune system dysfunction, and endocrine disruptors [22]. Thus, even modest reductions in PFAS levels could lead to meaningful improvements in public health, potentially reducing the burden on healthcare systems. Economically, the benefits of mitigating PFAS contamination extend beyond improved health outcomes. Obsekov et al. quantified the economic costs attributable to legacy PFAS exposures in the United States for 2018, estimating a lower bound of $5.52 billion in PFAS-related disease costs—with sensitivity analyses suggesting overall costs could be as high as $62.6 billion [23]. These figures underscore the substantial economic implications of regulatory inaction. In this way, if the legislative measures observed in our study contributed to the decline in PFAS concentrations, they offer not only environmental and health benefits but also economic advantages for Californians.

The public health and economic implications of PFAS underscore the broader societal value of robust environmental regulations. While our study focuses on the quantitative assessment of PFAS levels, understanding these downstream benefits further justifies the need for continued and enhanced policy efforts to address PFAS contamination. Building upon the findings of this research, future studies should consider several avenues for further investigation. Researchers may explore the specific mechanisms through which legislative changes influence PFAS contamination levels in drinking water. This investigation could involve in-depth analyses of compliance with MCLs, the effectiveness of monitoring efforts, and the impact of regulations on industries contributing to PFAS pollution. Additionally, it is crucial to consider identifying and regulating PFAS as a class. Currently, estimates of the number of PFAS substances vary. According to the U.S. EPA’s CompTox Chemicals Dashboard, there are nearly 15,000 recognized PFAS compounds [24]. However, other studies suggest that if a broader structural definition is applied—one that includes any chemical containing a –CF_2_– group—the number of PFAS could potentially approach 7 million [25]. This discrepancy, which reflects ongoing debate about what should be considered a “true” PFAS, underscores the challenges in regulating such a diverse group of chemicals and highlights the need for a unified, comprehensive approach. Furthermore, future studies should delve into the socioeconomic implications of PFAS contamination, with a particular emphasis on environmental justice communities. Examining disparities in exposure, access to clean water, and the effectiveness of remediation efforts can inform more effective and targeted policy interventions. Ultimately, our study suggests that the multifaceted approach taken by California between the pre-legislation period (2017) and post-legislation period (2023) can serve as a model for other regions facing similar challenges. By quantifying the impact of California’s legislative measures, this study demonstrates how combining contaminant thresholds, targeted financial allocations for water treatment, and product-level chemical restrictions may accelerate reductions in PFAS levels within public water systems. These results provide a rare, policy-specific benchmark for other industrialized nations considering enforceable drinking water limits and offer a practical template for countries struggling with PFAS contamination.

## Conclusions

The presence of PFAS contamination in California’s public water systems, affecting millions of residents, has emerged as a critical environmental and public health issue. PFAS contamination poses significant health risks, and its pervasiveness demands immediate attention. In response to this challenge, California introduced a multifaceted legislative framework to address PFAS contamination. The research conducted in this study reveals a significant decline in PFAS contamination levels in California’s public water systems following the enactment of legislation. While the results suggest a positive impact of the legislation on reducing PFAS contamination, there is more work needed to understand the full impact. Nevertheless, there is broad agreement that addressing PFAS contamination is imperative, and California’s legislative efforts serve as a valuable case study in this regard.

## Data Availability

All Python code used in this study is available in the Mitigating-PFAS-Contamination repository hosted on GitHub [26].
